# Stronger genetic differentiation among within-population genetic groups than among populations in Scots pine provides new insights into within-population genetic structuring

**DOI:** 10.1038/s41598-024-52769-y

**Published:** 2024-02-01

**Authors:** Darius Danusevičius, Om P. Rajora, Darius Kavaliauskas, Virgilijus Baliuckas, Algirdas Augustaitis

**Affiliations:** 1 Vytautas Magnus University, K. Donelaičio Str. 58, 44248 Kaunas, Lithuania; 2 Faculty of Forestry and Environmental Management, University of New Brunswick, PO Box 4400, 28 Dineen Drive, Fredericton, NB E3B 5A3 Canada; 3 Lithuanian Research Centre for Agriculture and Forestry, Forestry Institute, Liepu Str. 1, 53101 Kaunas Reg., Lithuania

**Keywords:** Ecological genetics, Forestry

## Abstract

We investigated the presence of spatial genetic groups within forest tree populations and determined if the genetic divergence among these groups is greater than that between populations using Scots pine (*Pinus sylvestris*) as a model species. We genotyped 890 adult trees of Scots pine in six natural populations in Lithuania at 11 nuclear microsatellite loci. We used a Bayesian clustering approach to identify the within-population genetic groups within each of the six populations. We calculated the differentiation indexes among the genetic groups within each population and among the six populations by ignoring the genetic groups. The Bayesian clustering revealed 2 to 6 distinct genetic groups of varying size as the most likely genetic structures within populations. The genetic differentiation indexes among the genetic groups within populations were nearly tenfold greater (*F*_ST_ = 0.012–0.070) than those between the populations (*F*_ST_ = 0.003). We conclude on the existence of markedly stronger structuring of genetic variation within populations than between populations of Scots pine in large forest tracts of northern Europe. Such genetic structures serve as a contributing factor to large within population genetic diversity in northern conifers. We assume that within population mating in Scots pine is not completely random but rather is stratified into genetic clusters. Our study provides pioneering novel key insights into structuring of genetic variation within populations. Our findings have implications for examining within-population genetic diversity and genetic structure, conservation, and management of genetic resources.

## Introduction

It is widely acknowledged that genetic diversity of forest tree populations is essential for strengthening forest resilience and sustainability, especially under rapidly changing climate^1–4^. The increasingly stronger environmental pressure requires a deeper understanding of genetic diversity and its structuring within and between forest tree populations to support sustainable forest management practices and conservation of genetic resources strategies e.g.^5–7^.

In forest trees, structuring of genetic diversity is influenced by evolutionary genetic processes such as genetic drift, selection, geneflow and mutation. Demographic stochasticity such as survival, reproduction as well as mating patterns can also affect population dynamics and genetic structure (e.g. Ref.^8^). The effects of these forces on genetic structuring of forest tree populations were well studied. However, most of these studies were primarily designed to study the among population differentiation and the within-population sampling was often too sparse for an appropriate investigation of the within population genetic structures^9^.

Most of the molecular marker-based studies in forest trees revealed weak among population genetic differentiation even on a geographically broad scale^9–17^ with most (≥ 90%) of the genetic diversity residing within populations. The phenomenon of weak population differentiation in open-pollinated conifers is explained by the existence of region-wide panmictic groups with a strong geneflow within and between these groups^16,18,19^. A vast within population genetic diversity in open-pollinated confers is also maintained and enriched by extensive long distance gene dispersal/geneflow^9,20,21^ as well as selection against inbreds at an early stage^20,21^.

The within-population spatial genetic structure (SGS) plays a key role in shaping the genetic diversity and inbreeding levels of future generations e.g.^22–24^. Knowledge of within-population SGS can also reveal the effects of primary evolutionary and ecological genetic processes operating in natural plant populations^25–28^. Therefore, SGS has been considered as a focal point for population genetics investigations. The SGS studies showed that in natural tree populations the genetic structures arise largely because of a common genetic background, and restricted pollen and seed dispersal^29–33^. The tree species with heavy fruits and sackless pollen (beech, oak) have a stronger SGS than the species with winged light seeds^22,34,35^. Therefore, SGS over 30 to 40 m was common for adult trees of oak (*Quercus*) species^36,37^, beech (*Fagus*) species^30,34,38–42^ and ash (*Fraxinus*) species^43^. Several studies showed that SGS depends on species-specific population structure and forest regeneration method^38,44,45^. A common feature of the earlier SGS studies was focusing on a single or a few neighboring populations. Such study design, however, is least suitable for finding reliable estimates of the among population genetic differentiation.

The previous studies on within-population structuring of genetic diversity in forest trees and other plants were using autocorrelation and/or kinship SGS analysis approaches without trying to estimate the strength of differentiation of genetic structures within populations (review of SGS methods^46^). The autocorrelation and kinship based SGS studies reveal the extent of SGS over a distance and do not reveal the existence of spatial genetic groups, their membership and genetic divergence. Also, most studies on the genetic structuring in forest trees were conducted either by having many populations but with low sample size or by having large sample sizes but only a few and adjacent populations. If sampling is too sparse or marker loci numbers are low, within-population genetic structuring may not be captured in full detail and may be underestimated^30,47^. For an objective comparison of the magnitude of genetic differentiation within populations versus between populations, within-population SGS should be studied for the same populations representing a geographical range by using a large sample size within populations. However, we could not find any such study reported in forest trees or other plants. If the studies focused on SGS estimates, they usually lacked a representative geographical range. On the other hand, if the aim of studies was on to examine geographical (population) genetic structure and differentiation, the population sample sizes were insufficient for proper detection of the within population genetic structures.

Bayesian clustering approaches may efficiently identify genetic groups and individuals belonging to specific genetic groups within populations, especially in complex spatial structures within forest tree populations^48,49^. For instance, the genetic groups may be intermixed or form overlapping irregular groups, where the linear autocorrelation approach may not be the most efficient. It would also be interesting to untangle the complex factors leading to a weak among population differentiation in widespread wind-pollinated forest tree species. Geneflow may not be the single factor reducing among population differentiation especially within a relatively smaller forest-rich region with no sharp adaptive gradients, such as Lithuania. Theoretically, widely distributed forest trees have large effective population sizes (e.g. Ref.^9^). Thus, genetic drift may be discarded as having a significant effect on the formation of within population genetic structures. However, over time, phenology-based structures within populations may lead to reduced genetic diversity within genetic groups in a population but high genetic diversity of a population itself^24^. Furthermore, this could also to some degree level out the genetic differences between populations, because geneflow may connect some within-population genetic groups more than with others.

Scots pine (*Pinus sylvestris* L.) serves as a good model species to examine fine-scale genetic structure within populations and compare the magnitude of within-population and interpopulation genetic divergence. Scots pine is a wide-ranging, wind-pollinated autochthonous conifer forming continuous forest tracts in Eurasia^50,51^. It is an outcrossing species with high levels of genetic diversity, most of which resides within populations, with a weak inter-population genetic differentiation^9^. Scots pine has a large effective population size^9^. Several studies have reported weak to significant SGS in managed or unmanaged Scots pine populations using autocorrelation and/or kinship analysis^52–54^. We examined genetic diversity and SGS in old-growth unmanaged (OGU) and second-growth managed (SGM) populations of Scots pine from different parts in Lithuania^55^. Significant autocorrelation and kinship-based SGS was observed in all populations with unmanaged populations having a significantly stronger SGS and larger genetic neighborhood size than the managed populations, but the among-population genetic differentiation was very weak. These observations provided a framework for more in-depth investigation of within-population structuring of genetic variation in the same populations.

In the present study, our objective was to study the fine-scale genetic structuring within Scots pine populations more in-depth than commonly done. We aimed to identify and characterize the within-population genetic structuring and compare the magnitude of genetic differentiation among these within-population genetic structures (groups) with the magnitude of among-population genetic differentiation in Scots pine. To account for what was lacking in most of the published genetic structure studies in forest trees, we used large sample sizes and fine-grid sampling in six natural Scots pine populations located within a geographical area devoid of sharp adaptive gradients in Lithuania. Such a strategy allows eliminating the already well-studied effects of natural selection from the complex model of genetic differentiation in populations of northern conifers.

We tested the hypothesis that within-population genetic structures (groups) are more strongly differentiated than the populations. Our idea for the existence of genetically diverse genetic groups within populations arises from an assumption that the trees within populations intermate within discrete clusters following cluster-specific flowering phenology^24,56^. If true, it could lead to a further hypothesis of prevalence of stratified over random mating systems within populations of forest trees. As phenology is strongly associated with adaptability in northern conifers (e.g. Ref.^57^), such new knowledge could contribute to understanding the adaptation processes in northern conifers.

## Results

### Within-population genetic diversity and geographical structuring

The frequency of null alleles was below 0.07 for all the loci, except for the locus *PtTX4011* with the null allele frequency of 0.17. All the 11 SSR loci were polymorphic with 4 to 34 alleles at a locus, yielding a highly variable data set for estimating the genetic structure and differentiation (Table 1). The EST-SSR loci were less polymorphic (especially *Psyl18* and *Psyl25*) with a lower between-population genetic differentiation than the genomic SSR loci (Table 1). For this reason, it is likely that in our study, the multilocus mean values of the observed and expected heterozygosities were below the common range expected for northern coniferous species. The genetic diversity parameters were similar for all six populations (Table 2). The observed heterozygosity (Ho) varied among the populations from 0.57 to 0.60, and the expected heterozygosity (He) varied between 0.59 and 0.61 (Table 2). The inbreeding coefficient (*F*_IS_) was close to zero for all populations and varied between 0.009 and 0.074 (Table 2).

**Table 1:** Locus-wise number of alleles, allelic range (most frequent allele and its frequency in %) and frequency-based genetic differentiation indices among the six Scots pine populations.

Locus	Allele range, bp	Repeat size, bp	A	H_o_	H_e_	Theta (*F*_ST_)	R_ST_	pG-test
EST-SSRs								
* Psyl16*	198–224 (202/25%)	2	8	0.75	0.81	0.001 ns	0.002	0.0418
* Psyl18*	285–312 (300/95%)	3	7	0.09	0.09	0.001 ns	− 0.001	0.6266
* Psyl2*	181–228 (208/78%)	2	8	0.35	0.35	− 0.001 ns	0.003	0.6527
* Psyl25*	218–236 (221/99%)	3	4	0.01	0.01	0.001 ns	0.006	0.0080
* Psyl42*	167–179 (173/39%)	2	5	0.68	0.69	0.001 ns	− 0.002	0.6003
* Psyl57*	191–210 (200/62%)	3	8	0.56	0.57	− 0.001 ns	0.001	0.2852
Genomic SSRS								
* PtTX4001*	201–237 (217/43%)	2	14	0.74	0.74	0.001 ns	0.003	0.0018
* PtTX4011*	195–283 (261/54%)	2	8	0.50	0.65	0.004	0.008	0.0001
* Spac11.4*	129–169 (139/25%)	2	17	0.87	0.85	0.005	0.002	0.0001
Spac12.5	121–195 (155/9%)	2	33	0.94	0.94	0.002	0.002	0.0001
Spac7.14	175–259 (215/6%)	2	33	0.91	0.95	0.002	− 0.001	0.0001
Mean			13.2	0.58	0.60	0.002	0.001	0.0001
CI 95%						0.001–0.003		

The abbreviation “ns” indicates the values of differentiation index Theta with bootstrapped standard error reaching 0 value and considered as not significantly different from 0. R_ST_ is R-statistics^58^.

pG-test is the p-value exact differentiation tests among the six populations based on allele frequencies (permuting individuals between populations, FSTAT). All the parameters were calculated for all 890 trees sampled.

**Table 2:** Age, sample size and genetic characteristics of the sampled populations.

Name of the population (ID)	Age class	Sample size	A	Ho	He	*F*_IS_
Azvintis (AZ2)^1^	100–150	203	12.6	0.59	0.61	0.032
Juodkrante (JUO)^1^	200–250	84	11.3	0.59	0.62	0.042
Punia (PUN) ^1^	200–250	196	13.2	0.57	0.61	0.074
Marcinkonys (DZ1)^2^	60–80	156	11.9	0.60	0.60	0.009
Azvintis (AZ1)^2^	100–150	119	12.0	0.57	0.61	0.064
Jonava (JV3)^2^	60–80	132	11.7	0.59	0.59	0.028

Age refers to the age class of Scots pine trees in the overstory that was sampled for the genotyping within ca. 1 ha size sample plots at the grid of 8 × 8 m within each population. Sample size is the number of genotyped trees. A is the mean number of alleles per locus. H_o_ is the observed, H_e_ is the expected heterozygosity; *F*_IS_ is the inbreeding coefficient.

^1^Old growth unmanaged stands (OGU). Strict nature reserves since 1950. Silvicultural treatment before 1950 is unlikely.

^2^Second growth managed populations (SGM) managed by standard silvicultural treatments since age of 10.

**Figure 1:**
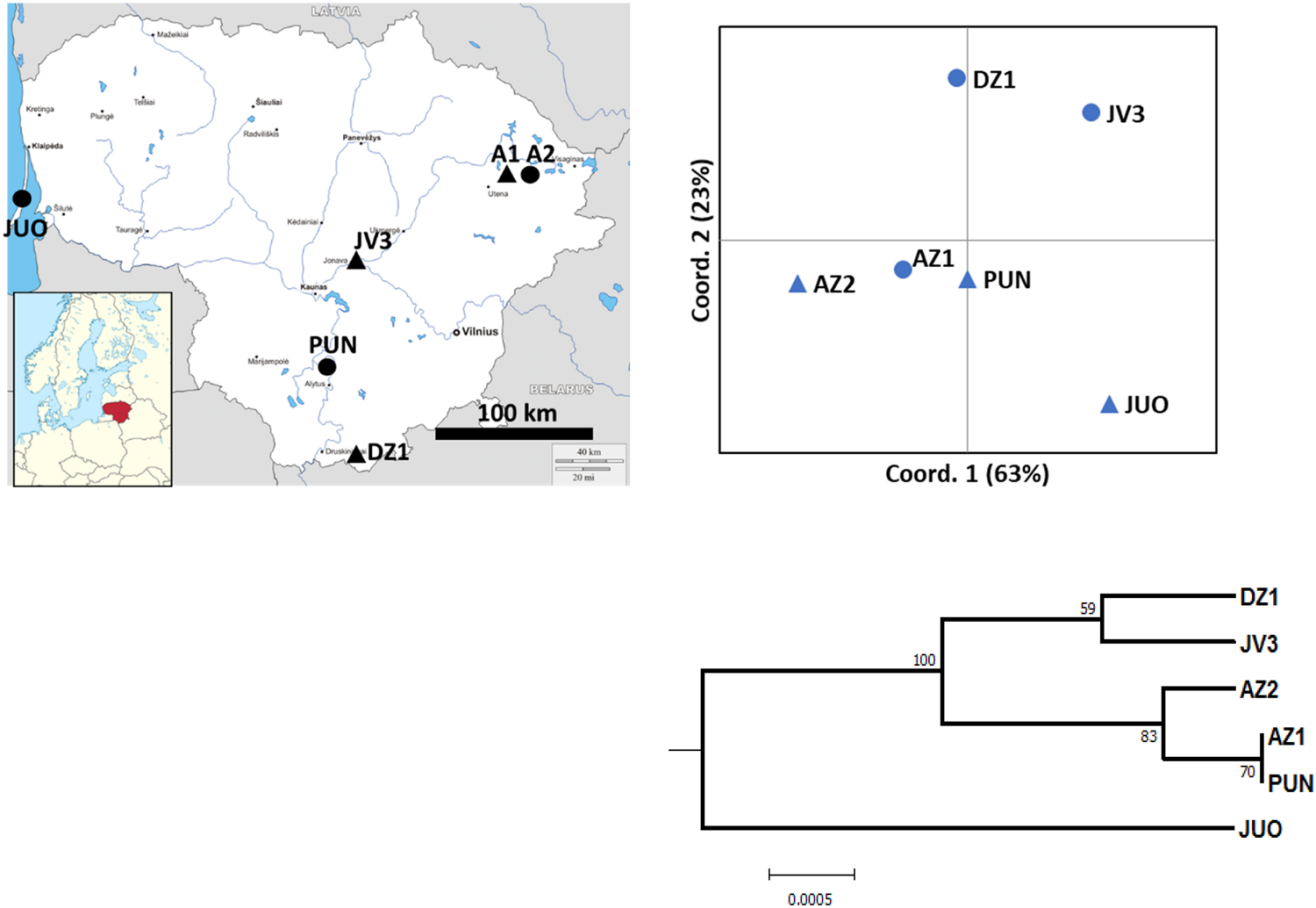
Upper left: location of the sampled populations: the old growth unmanaged populations are marked by filled circles and second growth managed populations are marked by triangles. Upper right: ordination of the populations on principal coordinates 1 and 2 based on codominant genotypic distance calculated from the SSR data; the percentages at the axis titles indicate the percentage of variation explained by the principal coordinates 1 and 2. Bottom right: UPGMA dendrogram based on Nei’s^59^ genetic distance (10 000 bootstraps over loci), the numbers at the nodes indicate the percentage of positive boots).

The PCoA plot of the six populations based on the first two principal coordinates (explaining 86% of the total genetic variation) revealed no strong geographical structuring (Fig. 1). However, some geographic tendencies were observed: the two neighboring north-eastern populations AZ1 and AZ2 clustered nearby and the most geographically outlying seaside JUO population was the most differentiated (Fig. 1). The UPGMA clustering supported the geographical clustering pattern found by the PCoA analysis (Fig. 1). The bootstrapped percentages of the UPGMA dendrogram nodes exceeded 58%, indicating a statistically reliable clustering structure (Fig. 1).

### Within-population genetic groups

**Figure 2:**
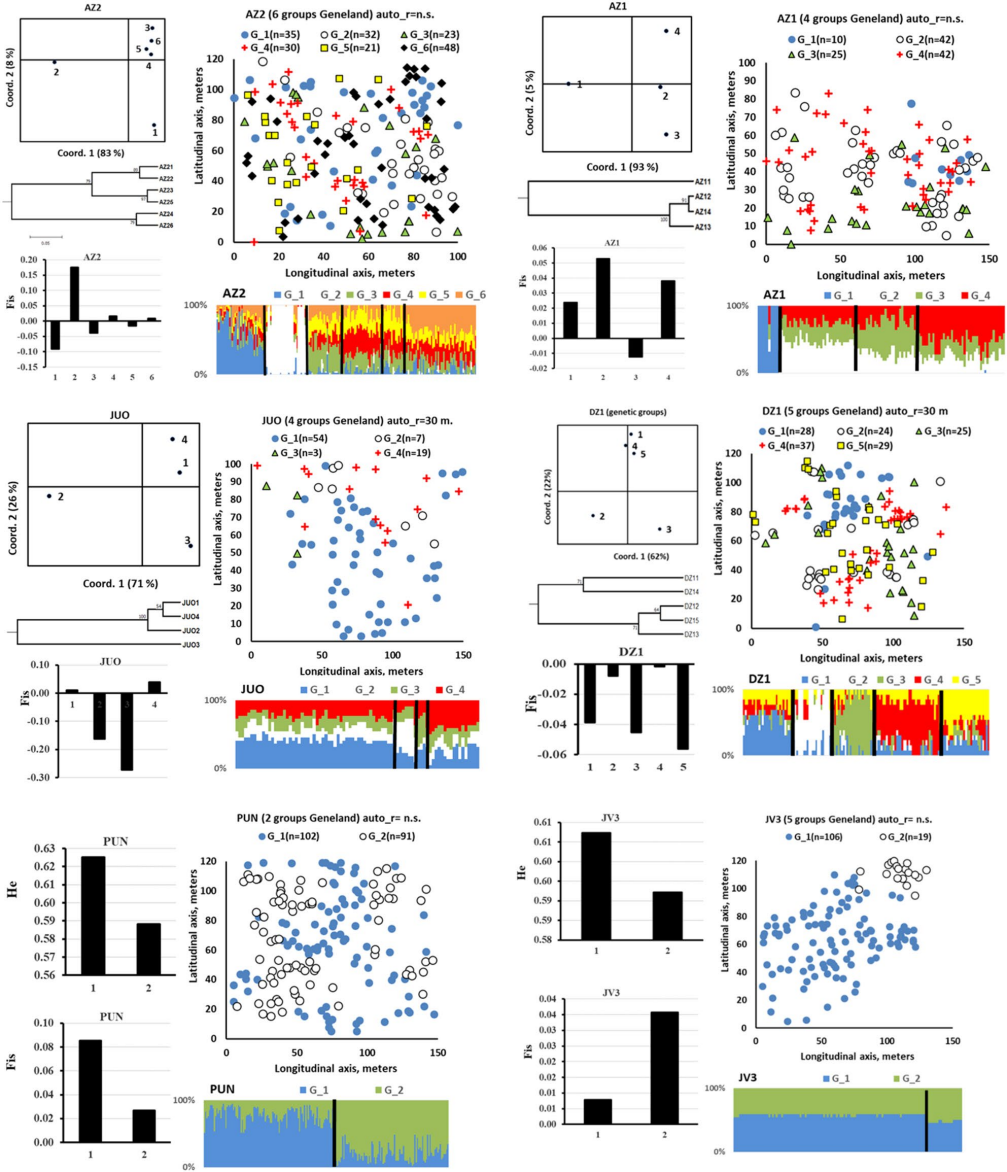
Spatial arrangement of the GENELAND genetic groups within the old growth unmanaged (left column) and second growth managed populations (right column). The X and Y axes indicate the actual size of the sample plots in meters. The symbols mark the actual location of individual trees. Different symbols define the GENELAND genetic groups. The sample size of each genetic group is given at the genetic group ID in the legend of the scatter plots. The extent of the largest spatial cluster size with significant kindship coefficient from the spatial autocorrelation analysis carried out in the present populations by Danusevičius et al.^55^ is given at the top of the scatter plots. Below each spatial plot, the GENELAND histograms of the genetic group membership coefficients (in %) are given. To the left of each spatial plot, the differentiation among the GENELAND genetic groups is shown by Principal Coordinates (PCoA) plots based on the *F*_ST_ distance and UPGMA dendrograms based on the Nei’s standard genetic distance (10 K bootstraps by D_ST_^59^). Plots of the multilocus mean *F*_IS_ values of the GENELAND genetic groups are also given. For the stands with two genetic groups (clustering is unfeasible for two groups) only H_e_ plots are added.

The GENELAND Bayesian clustering indicated a most likely genetic structure of 2 to 6 genetic groups within each of the six populations analyzed (Fig. 2). The 10 replicated GENELAND runs revealed the following within-population structures: for AZ1, 3–5 genetic groups (most frequent 4 groups); for DZ1, 5 to 7 genetic groups (most frequent 5 groups); for JV3, 1 to 3 genetic groups (most frequent 2 groups); for AZ2, 5 to 7 genetic groups (most frequent 6 groups); for JUO, 4 to 6 genetic groups (most frequent 4 groups) and for PUN, 1 to 2 genetic groups (most frequent 2 groups). The most frequent number of genetic groups over the 10 replicated runs in each population was chosen to be displayed in Fig. 2. A visual examination of the spatial location plots in Fig. 2 indicates a tendency for genetic group members to form spatial clusters especially in the two SGM populations DZ1 and JV3 and OGU population JUO genetic groups 2, 3, 4 were present in a single section of the sample plot.

The robustness of the genetic group structure is evident from the bar plots of the GENELAND group membership coefficients of the individual trees (Fig. 2 at the bottom of each figure section). Based on these bar plots, the strongest genetic structure was detected in two OGU populations AZ2, PUN2 and one SGM population DZ1 and the weakest in the SGM population JV3 (compare the least shared individual membership coefficients among the GENELAND genetic groups in the bar plots in Fig. 2).

The managed SGM population AZ1 contained a lower number of genetic groups than the nearby located unmanaged OGU population AZ2 (Fig. 2). Both AZ1 and AZ2 are naturally born populations sharing the local genepool. Therefore, it is likely that the systemic tending in AZ1 removed some of the genetic groups, basically leaving a relatively weaker genetic structure of genetic groups as compared to six genetic groups in AZ2 (Fig. 2). The robustness of the genetic structuring in OGU AZ2 was indicated by the quite uniform size of two strongly differentiating genetic groups with relatively high group membership coefficients (Fig. 2, the two leftmost genetic groups in AZ2 plot).

### Genetic characteristics of the within-population genetic groups

#### The OGU populations

In population AZ2, genetic groups 1 and 2 stand out due to their remarkably consistent GENELAND genetic group assignment coefficients, with group 2 exhibiting the least variability (Fig. 2). Additionally, group 2 displays the highest and group 1 the lowest inbreeding coefficient among the genetic groups in AZ2 (Fig. 2). In the JUO population, a single genetic group consisting of 54 trees was observed to be the dominant group. In the PUN population, two distinct genetic groups of comparable size were identified, but they exhibited varying levels of inbreeding.

#### The SGM populations

In contrast to the unmanaged OGU AZ2 population, most of the genetic groups in the AZ1 population exhibited positive inbreeding coefficients (Fig. 2). In AZ1, the genetic groups were generally genetically homogeneous, except for a distinct and spatially cohesive genetic group 1 (Fig. 2). Among the SGM populations, the DZ1 population contained the strongest genetic structures with 5 discrete almost equally sized genetic groups (Fig. 2). Furthermore, the genetic groups in DZ1 exhibited a clear structure, divided into two main clusters (the histogram in Fig. 2). Notably, groups 2 and 4 showed markedly lower inbreeding coefficients than the remaining genetic groups in DZ1. In contrast to its SGM counterparts, the JV3 population contained a rather homogenous structure with 2 genetic groups with different inbreeding levels, where a smaller group 2 clustered at a single spatial spot (Fig. 2).

Comparison of the genetic diversity parameters among the within-population genetic groups showed a marked variation in allelic diversity and observed heterozygosity estimates (Table 3). For instance, in AZ2, the groups 1 and 2 being of similar size varied markedly in observed heterozygosity and inbreeding (*F*_IS_) values (Table 3). Also, for other populations, the genetic diversity parameters varied among the genetic groups markedly, indicating a significant heterogeneity in genetic diversity and inbreeding levels among genetic groups within populations (Table 3).

**Table 3:** Comparison of the genetic diversity parameters among the genetic groups within each of the populations studied.

Population and genetic group	Number of trees	A	A_R_	A_e_	H_o_	H_e_	*F*_IS_
Old growth unmanaged populations							
AZ2_1	35	5.9	5.1	4.7	0.69	0.64	− 0.07
AZ2_2	32	6.1	5.1	5.8	0.56	0.64	0.17
AZ2_3	23	5.5	5.3	4.8	0.65	0.64	− 0.02
AZ2_4	30	6.5	5.9	5.3	0.64	0.67	0.06
AZ2_5	21	6.0	5.9	5.6	0.67	0.68	0.02
AZ2_6	48	6.7	5.4	5.7	0.64	0.65	0.02
JUO_1	54	7.8	6.0	5.4	0.60	0.61	0.04
JUO_2	7	3.6	na	3.2	0.58	0.55	− 0.07
JUO_3	3	2.4	na	2.5	0.61	0.57	− 0.08
JUO_4	18	5.6	5.6	4.7	0.57	0.61	0.10
PUN_1	102	10.3	11.7	5.9	0.57	0.63	0.09
PUN_2	91	10.5	10.4	5.3	0.57	0.59	0.04
Second growth managed populations							
AZ1_1	42	7.9	6.5	5.5	0.58	0.62	0.08
AZ1_2	25	6.1	5.9	4.6	0.57	0.58	− 0.01
AZ1_3	42	6.8	5.8	4.8	0.56	0.59	0.07
DZ1_1	28	7.5	6.2	4.9	0.61	0.60	− 0.03
DZ1_2	24	6.5	5.8	4.3	0.58	0.58	0.01
DZ1_3	25	6.9	5.7	3.9	0.53	0.51	− 0.04
DZ1_4	37	7.7	6.0	4.5	0.60	0.61	0.01
DZ1_5	29	8.5	6.9	5.1	0.64	0.61	− 0.04
JV3_1	104	11.1	7.6	5.8	0.59	0.61	0.03
JV3_2	19	7.5	7.5	4.1	0.56	0.59	0.06

A_R_ is the allelic richness, based on a minimum group sample size of 18 individuals. Other genetic diversity parameters are defined in Table 2.

**Figure 3:**
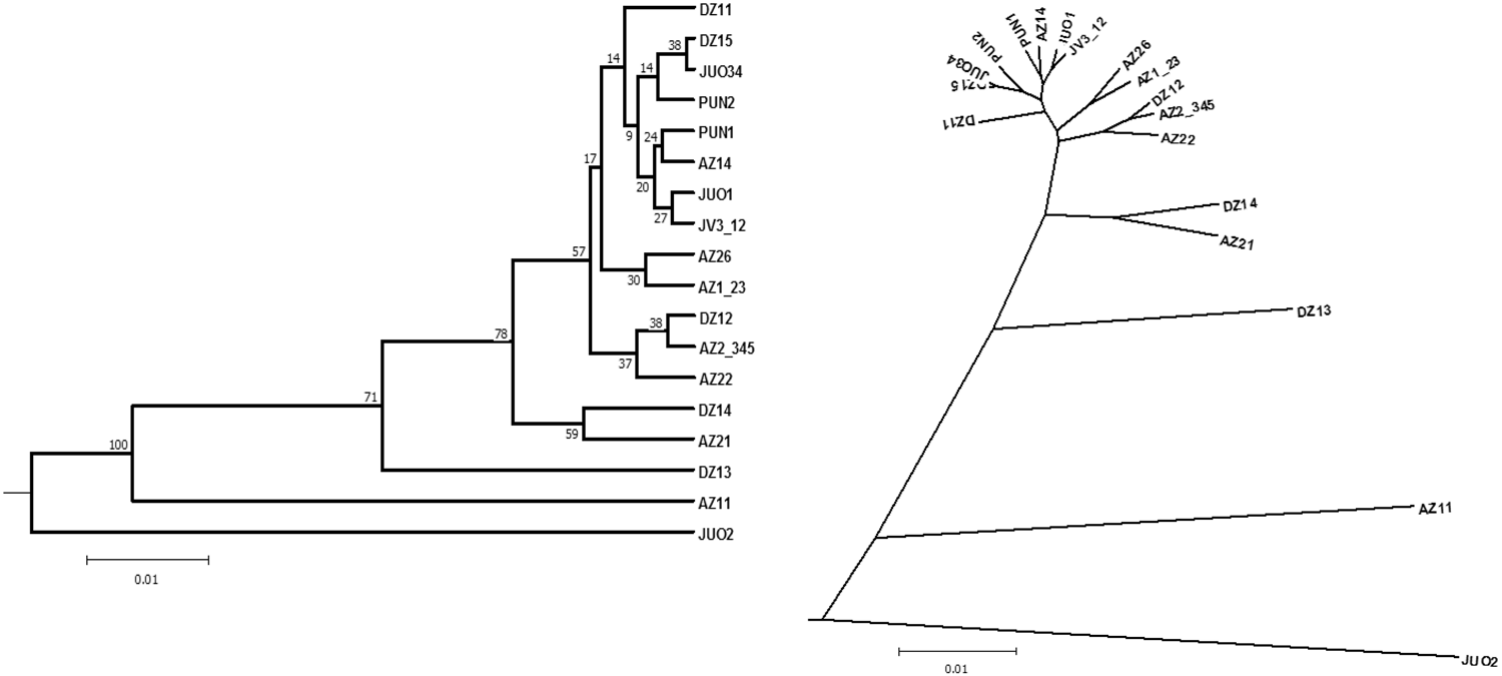
UPGMA dendrogram from clustering of the within-population genetic groups calculated by the Nei’s standard genetic distance (D_ST_, Ref.^59^). To reduce the complex structure of the dendrogram, several GENELAND groups with relatively low likelihoods for group assignment were pooled and noted as e.g., AZ2_345 (means groups 3, 4, 5 were pooled).

Clustering of the within-population genetic groups revealed the following most outlying single groups in our material (Fig. 3): JUO2, AZ1_1, DZ1_3, DZ1_4 and AZ2_1, the last two were grouped into a single cluster. The remaining within-population genetic groups formed two major clusters with a geographically mixed structure (Fig. 3). Noteworthy is that one of these two clusters contained genetic groups from each of the six populations (Fig. 3).

### Genetic differentiation

**Figure 4:**
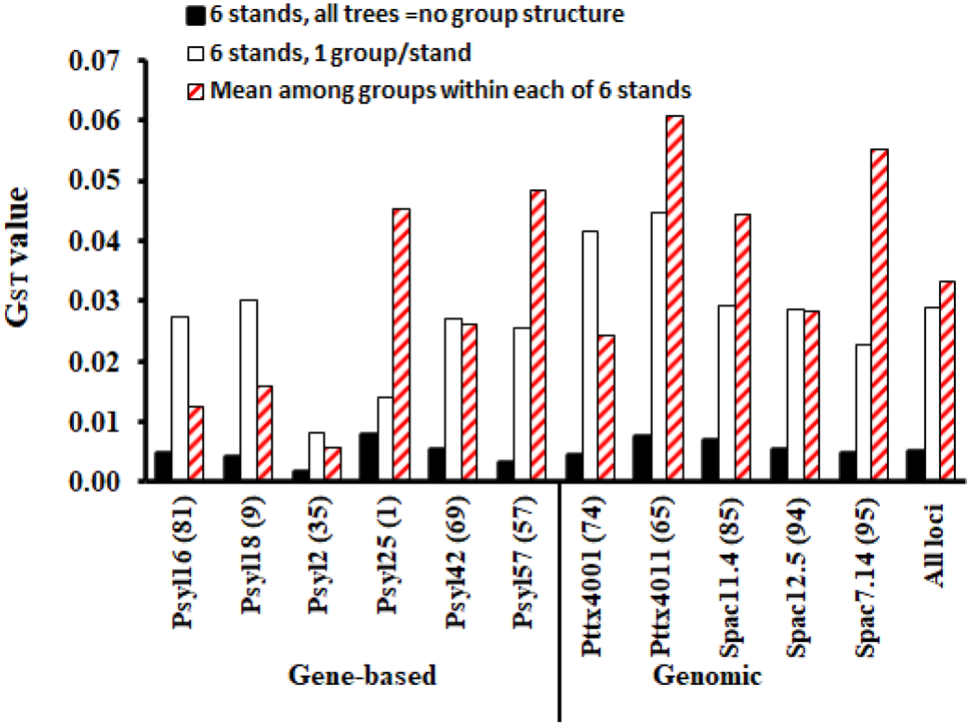
Allele frequency-based *F*_ST_ genetic differentiation index calculated (i) between the populations, with all trees with no information on within stand genetic structure (reflects geographical differentiation, filled bars in the plot), (ii) between populations, where each stand is represented by a single genetic group (white bars), (iii) among genetic groups within each of the 6 populations and then averaged for each locus (bars with the red diagonals). Numbers at the loci codes on the X axis show the expected heterozygosity values in %. “Gene-based” and “Genomic” indicate the microsatellite locus type.

The genetic differentiation tests among the six populations revealed weak and insignificant genetic differentiation indexes (Table 1). The locus-wise differentiation tests among the six populations showed a slightly stronger differentiation at the genomic SSR loci than at the EST-SSR loci (Table 1). Strikingly, for all loci, we found markedly greater genetic differentiation indexes among the within-population genetic groups than between the populations located in different parts of Lithuania (Fig. 4, Table 4). The locus-wise differentiation indexes among the genetic groups within the populations were especially high at the genomic SSR loci (Fig. 4). Another intriguing discovery was that the level of genetic differentiation observed between populations, each represented by a single genetic group (Fig. 4, white bars), was comparable in magnitude to the differentiation observed among the groups within the individual populations (Fig. 4, bars with the red diagonals). The R_ST_-based AMOVA revealed variance components of 0%, and 47% for among population, and among genetic group variance components, respectively (Table 5). The strongest among-group differentiation was observed in AZ2, AZ1 and DZ1 populations (Table 4, see the R_ST_ var. comps for pop.). The D_est_ index among the populations was tenfold lower than mean D_est_ among groups within populations for all populations except JV3 (Table 4).

**Table 4:** Results of the distance-based AMOVA and the allele frequency-based genetic differentiation tests between all 6 populations (first column) and the corresponding differentiation tests among the genetic groups within populations given for each population separately.

Genetic differentiation index	All 6 populations	AZ2 (6 groups)	JUO (4 groups)	PUN (2 groups)	AZ1 (4 groups)	DZ1 (5 groups)	JV3 (2 groups)
*F*_ST_(AMOVA)	0.003***	0.070***	0.031*	0.012***	0.048***	0.043***	0.002 ns
POP(VARCOM) %	0.30	7.0	3.0	1.0	5.0	4.0	0.2
*R*_ST_(AMOVA)	0.03***	0.63***	0.14*	0.02**	0.40***	0.29***	0.01 ns
POP(VARCOM) %	3.0	63	14	2.0	40	29.0	0.46
D_est_ (allele freq.)	0.003***	0.031***	0.081***	0.018***	0.055***	0.046***	0.002 ns
G_ST_ (allele freq.)	0.005***	0.035***	0.093***	0.009***	0.033***	0.040***	0.009 ns

The number of the genetic groups identified within each population is given in the Table heading. The significance levels for the p-value: *0.01–0.05; **0.001–0.01; ***< 0.001.

**Table 5:** Results of the hierarchical AMOVA to compare the levels of differentiation among the populations and among the genetic groups within populations calculated based on *F*_ST_ and R_ST_ distances.

Source	d.f	Var. comp (%) *F*_ST_	Var. comp. (%) *R*_ST_	Statistics	Value and significance
Among populations	5	0.0 (0%)	0 (0%)	F_RT_/R_RT_	− 0.01 ns/ − 0.18 ns
Among genetic groups	17	0.15 (4%)	6712 (47%)	F_SR_/F_SR_	0.04***/0.47***
Within genetic groups	1667	3.34 (96%)	7457 (53%)	*F*_ST_/R_ST_	0.03***/0.38***
Total	1689	3.49 (100%)	14,169 (100%)		

The *F*_ST_/R_ST_ distance-based differentiation indexes are as follows: F_RT_/R_RT_ among the populations in respect to total variation, F_SR_/R_SR_ among the genetic groups within populations and *F*_ST_/R_ST_ among the genetic groups in respect to total variation. The significance levels for the p-value: *0.01–0.05; **0.001–0.01; ***< 0.001.

## Discussion

We have for the first time demonstrated that varying number of spatial genetic groups of various sizes exist in Scots pine populations, and that the genetic differentiation among these genetic groups is much stronger (several folds) than among populations. The within-population genetic groups vary in their genetic diversity and inbreeding levels. If only one genetic group per population was used to examine the interpopulation genetic differentiation, the interpopulation genetic differentiation was comparable to that observed among the genetic groups within populations. We have provided pioneering novel key insights into the within population genetic structuring of a widely distributed coniferous tree species in northern Europe.

### Geographical structure, differentiation, and genetic diversity of populations

The weak geographical differentiation that we observed among the six populations (Fig. 1; Ref.^55^) indicates a strong homogenizing effect of geneflow between the Scots pine populations in Lithuania, which is consistent with other studies of wind pollinated conifers in northern Europe (e.g. Refs.^16,18,57^). The sample size of the populations in our study was large. Therefore, we can rule out the significant effect of sampling error on the levels of genetic differentiation observed in our study^55,60^. Population variation in flowering time over the photoperiodic and temperature gradients is an important factor leading to a significant genetic differentiation in northern conifers^61,62^. Apparently, the photoperiodic and temperature gradients in Lithuania are not strong enough to induce significant phenology gradients for Scots pine populations to counteract the homogenizing geneflow effects. Scots pine studies over a large geographical range reported higher genetic differentiation than in our study (14).

### Within-population genetic groups and their spatial arrangement

In our study, the populations contained similar genetic diversity levels, which allowed us for an objective comparison of the within-population genetic structures. Our study suggests that several genetic groups of various sizes exist within Scots pine populations even within such a small scale as one hectare. Furthermore, in contrast to populations, these genetic groups differ markedly in genetic diversity and inbreeding levels. Although the extent of SGS has been well studied in forest trees, we are not aware of any study reporting the existence of spatial genetic groups consisting of specific individuals.

The number of within-population genetic groups varied between the populations from 6 in AZ2 (OGU, north-eastern Lithuania) to 2 in JUO (OGU, seaside) and JV3 (SGM, central). Interestingly, in OGU AZ2 population of age 150–200, we found more genetic groups than in the adjacent SGM AZ1 population of age 80–100 (6 versus 4 groups, Fig. 2). It is likely that in unmanaged populations with a higher stocking more genetic groups remain, which otherwise may have been removed by tending treatments. This low intensity management could explain a stronger SGS in the OGU AZ2 population as was observed in the first study of this series^55^. This result also indicates that commercial tending may reduce genetic diversity by eliminating specific genetic groups rather than random individuals regardless of a genetic structure.

The spatial location plots of the genetic groups within the populations indicate an intermixed spatial arrangement of the genetically related individuals within the natural populations of Scots pine (Fig. 2). This result agrees well with earlier studies on SGS in conifers where spatially solid structures were rarely reported for open-pollinated widely distributed forest tree species^29,31,45,52^. The spatial autocorrelation analysis in our earlier study on the same material revealed significant spatial autocorrelation coefficients for 30-to-50-m distances^55^. These distances approximate well with the spatial arrangement of the genetic groups in the *in-situ* spatial location plots from the present study (Fig. 2). The SGM JV3 population with two spatially solid genetic clusters was an exception (Fig. 2). Such a situation may occur after an artificial refilling of open spaces in otherwise naturally regenerating populations. Therefore, the JV3-population-case may be treated as an exception.

The membership coefficients of assignment into the GENELAND genetic groups varied markedly among the genetic groups and between the populations (the GENELAND histograms in Fig. 2). This variation in the membership coefficients underscores the robustness and stability of the genetic structures within the populations. Presumably, the genetic groups consisting of individuals with high membership coefficients (such as groups 1, 2 in PUN, or groups 1, 2 in AZ2) represent strongly differentiated clusters of individuals within populations. However, when individuals exhibit roughly equal membership coefficients in multiple genetic groups, it suggests that they bear genetic signatures from several within-population genetic groups. This is indicative of a probable origin through inter-group mating, in contrast to intra-group mating.

The question arises why these genetic groups differentiate so strongly given no restriction for geneflow within the populations? Theoretically, this may be due to random genetic drift or a stochastic variability in demography, including phenology, survival, reproduction, and functional traits in the parental populations. Because the sampled populations are large and gene flow is strong, random genetic drift can be ruled out as being the main force causing significant differentiation of these within-population genetic groups. However, the variation in phenology timing may not be as stochastic as theoretically seems, but rather structured into groups of individuals with similar flowering synchrony within group (e.g. Ref.^24^). Obviously, individuals with similar flowering timing are likely to intermate and produce offspring for the subsequent generations. This assortative mating pattern, persisting over multiple generations, has the potential to drive genetic differentiation among distinct groups of individuals within populations to a degree observed in our study.

The initiation of divergent genetic groups within a population, as observed in PUN and AZ2, could potentially arise from a distant geneflow event that resulted in reduced flowering synchrony between the divergent genetic groups and the remaining population members. On the other hand, genetic groups that exhibit similar membership coefficients for assignment into multiple GENELAND genetic groups, such as groups 3, 4, and 5 in AZ2, may possess a greater phenological diversity and have the ability to mate with a larger number of individuals within the population. Stochastic natural regeneration may also lead to relatively weaker within population structures such as in JV3. Our data supports these considerations. Firstly, the genetic groups that possessed high GENELAND membership coefficients were genetically distinguished from the rest in the PCoA and the cluster analyses (for AZ2, groups AZ2_1 and AZ2_2; for AZ1, group AZ1_1; for DZ1, groups DZ1_2 and DZ1_3 in Fig. 2). These genetic groups were the ones that were outbranched in the UPGMA dendrogram in Fig. 3. Secondly, the genetic group AZ2_1 had more outbreeding levels than the rest (the *F*_IS_ graph in Fig. 2). Whereas the genetic group AZ2_2 had high inbreeding, markedly higher than the remaining groups in AZ2 population (Fig. 2). Presumably, the AZ2_2 genetic group may have originated from mating within a less outcrossing group of related trees, as discussed above. Such potential phenology association with genetic groups identified with molecular markers was found in *Fagus sylvatica* forest stands in Lithuania^24^.

### Genetic differentiation among and within populations

It is well documented that Scots pine, like other outcrossing wind-pollinated northern conifers, contains high levels of genetic diversity within populations and weak inter-population genetic differentiation^9,10,55^. This genetic diversity is continuously enriched by geneflow^18^. What we showed in our study is that this within-population genetic variation has a structure. More importantly, our study revealed that for Scots pine, the within-population genetic groups are markedly more strongly differentiated than the geographically distant populations are. What could cause such a strong genetic differentiation within the populations of Scots pine? Phenology observations on adult trees of Norway spruce visually distinguished 3 to 4 spring phenology groups in natural forests of Lithuania^63^. These phenology groups usually form distinct branching types and crown morphotypes in Norway spruce^64^. Similar phenology structuring as in Norway spruce could be assumed to exist in Scots pine populations. However, testing this hypothesis is not as straightforward for Scots pine as it is for Norway spruce. This is primarily because scoring phenology on tall Scots pine trees is challenging due to their tree morphology, with flowers located high up in the canopy. In support of our findings, several studies on within-population genetic structuring in pines forwarded the hypothesis of deviation from random mating due to grouping of reproductive individuals^65,66^. In contrast to our results, García Gil et al*.*^53^ could not distinguish more than a single genetic group with a SSR genotyping of 90 Scots pine trees sampled over a 25-ha area in northern Sweden. Obviously in García Gil et al*.*^53^ the sampling grid was too sparse for capturing significant genetic structures.

The within-population phenology-based groups may have a degree of intermating during the springs with high temperatures occurring over short time and, in this way, allowing some degree of overlapping in flowering time among otherwise phenologically distinct groups. In our study, this assumption is supported by groups of individuals sharing the GENELAND group membership coefficients for several genetic groups in almost equal proportions (Fig. 2). Phenology is an important adaptive trait for Scots pine in northern regions^62^. If these within-population genetic groups are phenology-based, then the genetic structuring based on SSR markers that we used may to some degree reflect the adaptive variation. Six of the 11 SSRs were from ESTs. In forest trees, phenology variation leads to differences in stem quality, manifesting by forking and spike knot defects^24^. As evidenced by high genetic diversity within most of the within-population genetic groups (including high heterozygosity and low inbreeding) in our study, it appears that significant accumulation of within-group coancestry does not occur in natural populations of Scots pine (Table 3). This suggests that such presumably stratified phenology-based mating may not lead to markedly elevated inbreeding or genetic drift in Scots pine.

Why was the genetic differentiation among the within-population genetic groups markedly stronger than the genetic differentiation between the populations? Geneflow may play a key role in the formation of these within-population genetic groups at the time of population regeneration. It is important to note that in the absence of pronounced environmental gradients, geneflow between Scots pine populations may exhibit stronger associations with only certain genetic groups within each population. As a result, if the populations share similar genetic groups (as observed in the upper portion of the NJ tree in Fig. 3), the "averaging" effect of the genetic exchange among these groups can potentially reduce the genetic differences between populations, leading to lower levels of differentiation between them. Strong support to this assumption is given in Fig. 4, when a single genetic group was randomly selected to represent each population, then the among-population differentiation values were high and comparable to those for within-population differentiation (Fig. 4).

## Conclusions and implications

We conclude that there is a markedly stronger structure of genetic variation within populations than between populations of Scots pine in large forest tracts of northern Europe. It is likely that mating of individuals within Scots pine populations does not follow a completely random pattern but may be stratified into genetic clusters. We provide key novel insights into finer-scale genetic structure within populations demonstrating the first time the existence of genetically differentiated spatial genetic groups of individuals within conifer populations. The existence of such genetically differentiated groups is likely a contributing factor for high within-population genetic diversity in conifers. Some programs, such as AMOVA, partition the total genetic variation into between populations and within populations. Naturally if populations have highly differentiated genetic groups, the between population genetic variance component will be low. These findings have implications for examining within-population genetic diversity and genetic structure, conservation, and management of genetic resources. The existence of genetically differentiated genetic groups should be considered when sampling populations for genetic diversity and population structure assessment. In the future, more in-depth studies should be undertaken for understanding the causes for existence of genetic groups of varying sizes and genetic diversity and inbreeding levels, for example examining the relationships between the phenology and genetic groups.

## Materials and methods

### The Scots pine populations and sampling

We studied three natural second-growth-managed (SGM) and three natural old-growth-unmanaged (OGU) populations of Scots pine in different parts of Lithuania (Fig. 1, Table 2). The sampled populations represent typical Scots pine-dominated large forest tracts on *Vaccinium and Vaccinium myrtilosum* site types in Lithuania. The population composition was 80 to 100% Scots pine with an admixture of Norway spruce (*Picea abies*). Within each population, we established rectangular sample plots of ca. 1 ha in size and randomly sampled the overstory Scots pine trees for the DNA genotyping (in some plots, we sampled almost all mature Scots pine trees). Geographical coordinates of each sampled tree were recorded with a GPS device to be used in the Bayesian clustering model (see below). Eighty-three to 203 trees were sampled per population, with a total of 890 Scots pine trees sampled in the six populations. Both SGM and OGU populations are of natural origin. The SGM populations were managed by a series of consistent tending treatments by promoting volume growth from the retained commercially superior trees. The OGU populations are nature reserves with no records of commercial management. A detailed description of the sampled populations is presented in Danusevičius et al*.*^55^.

### Genotyping

The sampled 890 Scots pine trees were genotyped at 11 nuclear microsatellite (SSR) loci (five genomic SSR and six EST SSR) as described in Danusevičius et al*.*^55^.

### Data analysis

#### Genetic diversity

We screened for null alleles by an algorithm estimating the excess of homozygotes implemented in the Micro-checker software ver. 2.2.3^67^. The commonly used genetic diversity parameters, allelic richness (*A*_R_, using rarefaction adjusted to the lowest sample size), and fixation index (*F*_IS_) estimates were calculated for individual populations and for individual within-population genetic groups (identified by Bayesian clustering, see below) by using GenAlEx soft. ver. 6.4^68^ and FSTAT soft. ver. 2.9.3.4^69^.

#### Population genetic structure and differentiation

The genetic structure within the sample plots was inferred separately in each population, by using a spatial Bayesian clustering approach implemented in the software GENELAND 2.0.10^70^. We preferred the GENELAND software for this propose over the other commonly used Structure software^71^, because the later bases the inferences on the genetic data alone, whereas GENELAND explicitly incorporates spatial location information of the genotyped individuals. Such clustering approach is a better option for the gene pools with a low level of differentiation, where the mating success depends on the spatial proximity^70^. The GENELAND settings were as follows: spatial model with correlated allele frequencies, maximum number of genetic groups (K) was 20, number of MCMC iterations was 1,000,000, thinning value of 1000, null allele filter was set on. In each run with K ranging from 1 to 20, all the individuals were assigned to K number of genetic groups by considering the SSR genotype and the spatial coordinates. Every individual was assigned to the genetic group with the highest membership coefficient. The most likely number of genetic groups (K) within each run with K ranging from 1 to 20 was identified by the highest posterior probability plots produced by GENELAND. For each population and K range 1 to 20, we ran GENELAND ten times and calculated the modal value of K over the ten repeated runs as the most likely number of genetic groups.

UPGMA and NJ clustering of the within-population genetic groups was carried out based on Nei’s^59^ genetic distances with POPTREE2 software^72^ by testing the significance of dendrogram branches with 10,000 bootstraps among the loci. Principal Coordinate Analysis (PCoA) was carried out to estimate the genetic relationships among the six populations as well as among the within-population genetic groups for each of the six populations with GenAlEx ver. 6.4 software^68^.

We calculated the allele frequency-based genetic differentiation indexes (G_ST_ and D_est_, GenAlEx ver. 6.4 software^68^): (a) among the six populations containing all the sampled individuals, (b) among the six populations containing a single randomly selected GENELAND genetic group to test cases with a reduced complexity of within-population genetic structuring, and (c) among the GENELAND genetic groups for each of the six populations separately. For comparison, we also used FSTAT ver. 2.9.3.2 software^69^ to estimate locus-wise Theta differentiation indexes (a version of *F*_ST_) and to perform the exact differentiation test (G-test^69^) among the six populations. The FSTAT Theta values are adjusted for unequal sample size^73^, where its 95% CI are obtained by bootstrapping over loci. If the CI values do not include the 0 value, the theta value is considered as significantly different from 0. For the exact differentiation test (G-test), the significance was tested by 10,000 permutations of alleles between samples and the proportion (p) of runs with randomly assigned alleles giving a larger G-test statistics than observed is considered as an indicator of significance (if p < 0.05 then differentiation considered as significant).

We also performed a hierarchical analysis of molecular variance (AMOVA) implemented in Arlequin soft. ver. 3.5.1.3^74^ to partition the total molecular variation into the following components: among populations, among genetic groups within populations, and within genetic groups. For the AMOVA, we used both allele identity (*F*_ST_-like option in Arlequin) and allele size (*R*_ST_-like option in Arlequin) differentiation indexes based the genetic distances and 1000 permutations for testing the statistical significance. The AMOVA calculates the Phi_ST_ statistics based on variance components which are analogous to Wright’s *F*_ST_.

### Statement on research involving plants

The authors comply with the IUCN Policy Statement on Research Involving Species at Risk of Extinction and the Convention on the Trade in Endangered Species of Wild Fauna and Flora.

## Data Availability

Data is available upon request to darius.danusevicius@vdu.lt.
